# Predicting Global Functional Outcomes Among Post-traumatic Epilepsy Patients After Moderate-to-Severe Traumatic Brain Injury: Development of a Prognostic Model

**DOI:** 10.3389/fneur.2022.874491

**Published:** 2022-05-30

**Authors:** Tingting Yu, Xiao Liu, Lei Sun, Ruijuan Lv, Jianping Wu, Qun Wang

**Affiliations:** ^1^Department of Neurology, Beijing Tiantan Hospital, Capital Medical University, Beijing, China; ^2^China National Clinical Research Center for Neurological Diseases, Beijing, China; ^3^Advanced Innovation Center for Human Brain Protection, Capital Medical University, Beijing, China; ^4^Beijing Institute of Brain Disorders, Collaborative Innovation Center for Brain Disorders, Capital Medical University, Beijing, China

**Keywords:** post-traumatic epilepsy, traumatic brain injury, global functional outcome, risk factor, prognostic model

## Abstract

**Objective:**

The development of post-traumatic epilepsy (PTE) following traumatic brain injury (TBI) is associated with unfavorable functional outcomes, and the global function of PTE patients might change dynamically overtime. Predicting the long-term functional outcomes of patients with PTE may help to develop accurate rehabilitation programs and improve their quality of life. Based on this, the objective of this study is to use clinical data to derive and validate a model for predicting the functional outcomes of patients with PTE after moderate-to-severe TBI.

**Methods:**

This study retrospectively analyzed 721 patients with PTE after moderate-to-severe TBI in the Epilepsy Centre, Beijing Tiantan Hospital, from January 2013 to December 2018. All patients had favorable global function as indicated by the Glasgow Outcome Scale-Extended (GOSE) at the time of their first late post-traumatic seizure (PTS) onset, and the 5-year global function after the first late PTS onset was chosen as the principal outcome of interest. To identify possible predictors for the global functional outcomes, univariate and multivariate logistic regression techniques were used. A prognostic model was established using these identified predictors, the internal validation with the bootstrapping method was performed, and the model was then visualized as a graphical score chart.

**Results:**

The 5-year global functional outcome of 98 (13.59%) patients was unfavorable, and the temporal lobe lesion was found as the strongest predictor of unfavorable outcomes. The final prognostic model also included the following other predictors: gender, age at TBI, multiple injuries, the severity of TBI, and latency of PTE. Discrimination was satisfactory with C-statistic of 0.754 (0.707 – 0.800), the goodness-of-fit test indicated good calibration (*P* = 0.137), and the C-statistic was 0.726 for internal validation. A graphical score chart was also constructed to provide the probability of an unfavorable 5-year global functional outcomes more readily.

**Conclusions:**

Clearer treatment strategies are essential to help ameliorate the global functional outcomes of patients with PTE. Our proposed prognostic model has significant potential to be used in the clinic for predicting global functional outcomes among patients with PTE after moderate-to-severe TBI.

## Introduction

Traumatic brain injury (TBI) is one of the leading causes of death and disability worldwide (1, 2), and there are approximately one million new cases of TBI annually in China alone (3). Previous large-scale studies have concluded that only about 40–50% of individuals achieve a favorable outcome 6 months after moderate-to-severe TBI (4, 5), and within 10 years after TBI, the long-term outcome may tend either to deteriorate or improve (6, 7). Pre-injury employment, white-collar work, and shorter post-trauma amnesia duration have been reported as prognostic factors for better long-term outcomes (7), while male gender, younger age, less severe TBI have shown good prognostic effects on long-term outcomes in several studies (7, 8), but no such prognostic effects were observed in other studies (6, 9). Crucially, however, the importance of rehabilitation programs for certain TBI patients has been highlighted for favorable outcomes (6).

Post-traumatic seizure (PTS) is one of the most common sequelae of TBI and is classified as immediate PTS (within 24 h postinjury), early PTS (between 1 and 7 days postinjury), or late PTS (more than 7 days postinjury) according to the time of occurrence, and recurrent unprovoked late PTS is referred to as PTE (10). The presence of PTE in particular has been found to develop in 4.2–53% of patients who suffer moderate-to-severe TBI (10–13). Since TBI most commonly occurs in young adults (3), who may survive for decades after their injury (14), TBI might in fact become a chronic health condition rather than an acute event (15), especially for those who develop PTE after TBI. Some authors have reported that the development of PTE following TBI is independently associated with unfavorable functional outcomes (16–19). Hence, predicting the long-term outcomes of patients with PTE may help clinicians to provide more personalized medical care and rehabilitation programs that can better improve these patients' quality of life.

During clinical admission of PTE patients, clinicians may pay more attention to the seizure outcome rather than the global functional outcome. Although many studies have focused on the global functional outcome of patients with TBI (5, 7, 20), few studies have systematically investigated the global functional outcome of patients with PTE as well. This lack of study prevents clinicians from effectively identifying patients whose global function prognoses were likely to deteriorate and from further developing clearer treatment strategies for them. Briefly, clinical management of PTE requires recognition of the heterogeneous endophenotypes associated with functional outcomes. Identifying the risk factors for functional decline in patients with PTE and establishing a simplified prognostic model for predicting the long-term functional outcome may therefore become quite useful in improving the therapy of PTE.

This study sought to describe global function changes up to 5 years after the first late PTS onset, identify predictors that significantly relate to 5-year global functional outcomes, and to develop a prognostic model that can be used at the clinic for predicting unfavorable functional outcomes among patients with PTE after moderate-to-severe TBI.

## Methods

### Study Participants

Clinical data of 2,862 patients who were diagnosed with PTE in the Epilepsy Center of Beijing Tiantan Hospital from January 2013 to December 2018 were retrospectively reviewed. All PTE patients meeting inclusion criteria were followed up continuously for at least 3 years in the clinic or by telephone, and all last-time follow-ups were completed between September 2019 and August 2021 at which time the score on the Glasgow Outcome Scale-Extended (GOSE) (21) was recorded to assess global functional outcomes. For each patient enrolled in this study, the total duration of case review plus continuous follow-up was a minimum of 5 years.

Inclusion criteria consisted of: (1) age 16–55 years at the time of TBI; (2) “moderate-to-severe” severity of TBI; (3) meeting the diagnostic criteria of PTE; and (4) having a favorable global function (GOSE 5 to 8) at the onset of the first late PTS. Excluded criteria were: (1) perinatal injury, febrile convulsion, or seizure prior to TBI; (2) pre-existing neurological disease, systemic metabolic disease, or major organ disease; (3) GOSE score not being able to be accurately recorded due to a lack of sufficient information; and (4) age >80 years at the time when the 5-year GOSE score is recorded.

The study was approved by the Ethics Committee of the Beijing Tiantan Hospital affiliated with the Capital Medical University of the People's Republic of China. The study was conducted in accordance with the Declaration of Helsinki, and all participants provided informed consent for the use of their medical records.

### Data Collection

Clinical data including demographic information, TBI details, the clinical condition of PTE (such as the presence of acute seizure, the latency of PTE, the type of seizure, seizure frequency, and the presence of status epilepticus [SE]), the electroencephalogram (EEG), the usage of antiseizure medications (ASMs), and patients' drug responses were collected, as mentioned in our previously published study (22).

The severity of TBI was judged based on neurological and imaging evaluations: moderate TBI was characterized by loss of consciousness or post-trauma amnesia lasting 30 min to 24 h, with or without skull fracture; and severe TBI was characterized by brain contusion, intracranial hematoma, loss of consciousness lasting ≥24 h, or post-trauma amnesia lasting ≥24 h (10). In addition, this study recorded severe TBI cases as “severe TBI with conservative treatment” or “severe TBI with surgical operation” (puncture drainage or decompressive craniectomy during the acute phase of TBI, with or without following cranioplasty operation) according to their courses of treatment. Lesions caused by TBI were also divided into temporal lobe (left/right) lesions or lesions outside the temporal lobe, and classified craniocerebral injuries as either a single injury or multiple injuries (22).

Acute seizure refers to a seizure that occurs within 7 days after TBI, including immediate PTS and early PTS. This study recorded the time interval between TBI and the onset of the first late PTS as the latency of PTE. Additionally, in accordance with the 2017 classification of the International League Against Epilepsy (ILAE) (23), the seizure type of PTE within the first 2 years of the course of PTE was recorded as generalized onset seizure, focal onset seizure, or mixed onset seizure, and the presence of SE of each individual within the first 2 years of the course of PTE was also recorded in accordance with the 2015 definition of SE by the ILAE as well (24). To enable the final model to play a predictive role in the early stages of the development of PTE, this study only included data from the first 2 years of PTE in the initial analysis.

For each patient, two neurologists (TTY and XL) reviewed the original EEG data or the EEG report and assessed the EEG as “normal EEG”, “abnormal background without epileptiform discharges”, or “epileptiform discharges”. The EEG was a randomly selected routine interictal EEG (20–40 min monitoring) during the outpatient visits. The usage of ASMs and the drug response for each individual were also recorded, and the development of drug-resistant epilepsy (DRE) was assessed by two neurologists (TTY and QW) according to the definition of DRE by the ILAE (25).

### Global Function

Scores on the GOSE were used for global functional outcome assessment (21) and were obtained through structured interviews (26). The GOSE is a global scale for functional outcomes that rates patient status into eight categories: 1, death; 2, vegetative state; 3, lower severe disability; 4, upper severe disability; 5, lower moderate disability; 6, upper moderate disability; 7, lower good recovery; and 8, upper good recovery (26). In this study, a GOSE score of 5–8 was defined as indicative of favorable global function and a GOSE score of 4 or less was defined as indicative of unfavorable global function. The GOSE at the onset of the first late PTS was recorded based on information recalled by the patients or their caregivers when the patient first visited our epilepsy center. At the last follow-up from September 2019 to August 2021, the 5-year GOSE of each patient was also recorded in the clinic or by telephone. All patients enrolled in this study were divided into two groups: patients with a 5-year GOSE score of 5–8, the favorable outcome group and patients with 5-year GOSE score of 4 or less, the unfavorable outcome group.

### Predictors

Eleven variables were analyzed as potential predictors of functional outcomes, including demographic characteristics (gender, age at TBI), TBI details (severity of TBI, lesion location, single, or multiple injuries), and PTE characteristics (the presence of acute seizure, latency of PTE, type of seizure, the presence of SE, EEG findings, and the development of DRE).

### Statistical Analysis

To carry out our statistical data analysis, SPSS 23.0 software (IBM Crop., Armonk, NY) and *R* version 4.1.1 software were used. Continuous data were transformed into mean ± SD or median and interquartile range (IQR), and numerical data were transformed into percentages. The Mann-Whitney *U*-test was used to compare continuous data and the χ^2^ or Fisher exact test was used to compare numerical data as appropriate. After this, univariate and multivariate logistic regression were performed to identify predictors significantly related to global functional outcomes. Predictors with *p* < 0.3 in the univariate logistic regression analysis were included in the initial multivariable logistic regression for further analysis, and the data were converted to adjusted odds ratios (ORs) with 95% CIs. A two-sided test with a *p* < 0.05 was deemed to be statistically significant.

### Development, Validation, and Presentation of the Prognostic Model

In the univariate and multivariate logistic regression analysis, two continuous variables, age at TBI and the latency of PTE, were converted into dichotomous variables. Age at TBI was recorded as < 30 years old (youth), or ≥ 30 years old (young and middle-aged, middle-aged). Previous studies have reported that the latency of PTE in most PTE patients is < 1 year, accounting for 60%−80% of all cases (11, 14, 27). Accordingly, the latency of PTE was classified as either < 12 months or ≥ 12 months.

After including all candidate predictors with *p* < 0.3 from the univariate logistic regression into the initial multivariable logistic regression, the nonsignificant predictors were then eliminated in a backward stepwise fashion and the final model we selected was the model with the minimum Akaike Information Criterion (AIC).

Next, the performance of this prognostic model was evaluated in terms of discrimination and calibration. Discrimination indicates whether the model can correctly distinguish favorable and unfavorable 5-year global functional outcomes and was measured by calculating the areas under the receiver operating characteristic curves (AUC) to form the C-statistic. An AUC of > 0.7 indicates acceptable discrimination. After calculating the discrimination, this study applied 1,000 bootstrap resamples to establish a calibration curve used to indicate whether actual outcomes agree with predicted risks, and this study evaluated calibration by using a goodness-of-fit test, where a *p*-value > 0.05 indicates good calibration. Finally, internal validation was also performed with the bootstrapping method.

For ease of use at the clinic, this study presented the prognostic model as a graphical score chart in a simplified, color-coded version (28). In this graphical score chart, predictors were cross-tabulated, and the probabilities of unfavorable 5-year global functional outcomes for each individual with values of each predictor were estimated in each cell. The cells of the chart were then colored into four groups, according to the ranges of the probabilities.

## Results

### Patients Characteristics

After retrospectively screening the clinical records of 2,862 patients diagnosed with PTE, 1,208 patients met the inclusion criteria mentioned above. Of all the 1,208 patients, 18 patients had the perinatal injury, febrile convulsion, or seizure prior the TBI, 87 patients had pre-existing neurological disease, 377 patients lacked sufficient information for GOSE records, 5 patients were more than 80 years old when the 5-year GOSE was recorded, and all of these patients were excluded, leaving us with a total of 721 patients with PTE after moderate-to-severe TBI for analysis. The percentage of patients who were male was 88.5%, with a median age of all patients at TBI of 26.0 years (IQR, 21.0–35.0), the median age at last follow-up of 38.0 years (IQR, 32.0–48.0), and median PTE course of 7.9 years (IQR, 6.6–8.9). The total rate of unfavorable outcomes (GOSE scores of 4 or less) 5 years later was 13.59% (98/721). The characteristics of all patients and the differences between the two groups are shown in Table 1.

**Table 1:** The favorable outcome group vs. the unfavorable outcome group comparison summary table.

	**Total (*n =* 721)**	**Favorable (*n =* 623)**	**Unfavorable (*n =* 98)**	***P*-Value^†^**
**Demographics**				
Gender (males, %)	638 (88.5%)	544 (87.3%)	94 (95.9%)	0.013*
Course of PTE (years, IQR)^a^	7.9 (6.6–8.9)	7.9 (6.5–9.0)	7.8 (6.8–8.9)	0.594
Age at TBI (years, IQR)	26.0 (21.0–35.0)	25.0 (21.0–34.0)	30.0 (23.0–42.3)	0.000**
Age at last follow-up (years, IQR)	38.0 (32.0–48.0)	37.0 (32.0–47.0)	38.0 (33.0–51.0)	0.094
Multiple injuries	383 (53.1%)	311 (49.9%)	72 (73.5)	0.000**
**Lesion location**				0.000**
Outside temporal lobe	291 (40.4%)	273 (43.8%)	18 (18.4%)	
Left temporal lobe	180 (25.0%)	141 (22.6%)	39 (39.8%)	
Right temporal lobe	250 (34.7%)	209 (33.5%)	41 (41.8%)	
**Severity of TBI**				0.000**
Moderate TBI	245 (34.0%)	227 (36.4%)	18 (18.4%)	
Severe TBI + C	120 (16.6%)	106 (17.0%)	14 (14.3%)	
Severe TBI + S	356 (49.4%)	290 (46.5%)	66 (67.3%)	
The presence of acute seizure	34 (4.7%)	27 (4.3%)	7 (7.1%)	0.223
Latency of PTE (months, IQR)^b^	12.0 (4.0–58.0)	12.0 (5.0–60.0)	7.0 (2.0–18.0)	0.000**
**Seizure type**				0.034*
Generalized onset	102 (14.1%)	85 (13.6%)	17 (17.3%)	
Focal onset	547 (75.9%)	482 (77.4%)	65 (66.3%)	
Mixed onset	72 (10.0%)	56 (9.0%)	16 (16.3%)	
The presence of SE	48 (6.7%)	38 (6.1%)	10 (10.2%)	0.130
**EEG fundings**				0.147
Normal	130 (18.0%)	106 (17.0%)	24 (24.5%)	
Abnormal background	100 (13.9%)	85 (13.6%)	15 (15.3%)	
Epileptiform discharges	491 (68.1%)	432 (69.3%)	59 (60.2%)	
The presence of DRE	122 (16.9%)	108 (17.3%)	14 (14.3%)	0.454

^†^Comparison between favorable outcome group and unfavorable outcome group; ^a^the time interval between the onset of first late PTS and the last-time follow-up; ^b^the time interval between TBI and the onset of first late PTS; *p < 0.05; **p < 0.01; continuous data were transformed into the median and interquartile range, numerical data were transformed into percentages.

*TBI, traumatic brain injury; SE, status epilepticus; EEG, electroencephalogram; DRE, drug-resistant epilepsy; severe TBI + C, severe TBI with conservative treatment; severe TBI + C, severe TBI with surgery; PTS, post-traumatic seizure; IQR, interquartile range*.

### Risk Factors for Functional Disability

All of the 11 tested variables had no correlation (absolute value of correlation coefficient < 0.3) with each other (Supplementary Figure S1). The univariate logistic regression showed that 10 of the 11 variables had a *P* < 0.3; the exception was the development of DRE (Table 2). All of 10 variables with *P* < 0.3 were entered into the initial multivariable logistic regression. After backward stepwise elimination, 6 variables remained in the final logistic regression model, with a minimum AIC of 520.24. These variables were gender, age at TBI, lesion location, single or multiple injuries, the severity of TBI, and latency of PTE (Table 3), and the multicollinearity was low (variance inflation factors < 5) for the final model (Supplementary Table S1).

**Table 2:** Univariate logistic regression of unfavorable 5-year global functional outcomes.

**Variable**	**OR**	**OR 95%*CI***	***P*-Value**
Gender (Female)	0.293	0.088–0.726	0.019*
Age at TBI (≥30.0 years)	1.988	1.294–3.059	0.002**
**Lesion location**			
Outside temporal lobe	Ref		
Left temporal lobe	4.195	2.348–7.759	0.000**
Right temporal lobe	2.975	1.686–5.445	0.000**
Multiple injuries	2.778	1.749–4.540	0.000**
**Severity of TBI**			
Moderate TBI	Ref		
Severe TBI + C	1.666	0.786–3.466	0.174*
Severe TBI + S	2.870	1.691–5.105	0.000**
The presence of acute seizure	1.698	0.665–3.812	0.228*
Latency of PTE (≥12.0 months)	0.397	0.254–0.614	0.000**
**Seizure type**			
Generalized onset	Ref		
Focal onset	0.674	0.384–1.238	0.184*
Mixed onset	1.429	0.663–3.071	0.359
The presence of SE	1.749	0.800–3.510	0.134*
**EEG findings**			
Normal	Ref		
Abnormal background	0.779	0.378–1.564	0.489
Epileptiform discharges	0.603	0.362–1.029	0.057*
The presence of DRE	0.795	0.419–1.410	0.455

*p < 0.30; **p < 0.01.

*TBI, traumatic brain injury; severe TBI + C, severe TBI with conservative treatment; severe TBI + C, severe TBI with surgery operation; PTS, post-traumatic seizure; SE, status epilepticus; EEG, electroencephalogram; DRE, drug-resistant epilepsy; OR, odds ratio; CI, confidence intervals; Ref, reference*.

**Table 3:** Multivariate logistic regression of unfavorable 5-year global functional outcomes.

**Variable**	**β-Coefficient**	**OR (95%*CI*)**	***P*-Value**
Intercept	−3.278	*NA*	*NA*
Gender (Female)	−1.130	0.323 (0.095–0.826)	0.035*
Age at TBI (≥ 30.0 years)	0.608	1.836 (1.162–2.905)	0.009*
**Lesion location**			
Outside temporal lobe	Ref		
Left temporal lobe	1.109	3.031 (1.626–5.817)	0.001**
Right temporal lobe	0.981	2.668 (1.482–4.970)	0.001**
Multiple injuries	0.606	1.834 (1.100–3.120)	0.022*
**Severity of TBI**			
Moderate TBI	Ref		
Severe TBI + C	0.415	1.514 (0.694–3.248)	0.289
Severe TBI + S	0.710	2.034 (1.156–3.724)	0.017*
Latency of PTE (≥ 12 months)	−0.667	0.513 (0.320–0.815)	0.005**

*p < 0.05; ** p < 0.01.

*TBI, traumatic brain injury; severe TBI + C, severe TBI with conservative treatment; severe TBI + C, severe TBI with surgery; OR, odds ratio; CI, confidence intervals; NA, not applicable*.

**Figure 1:**
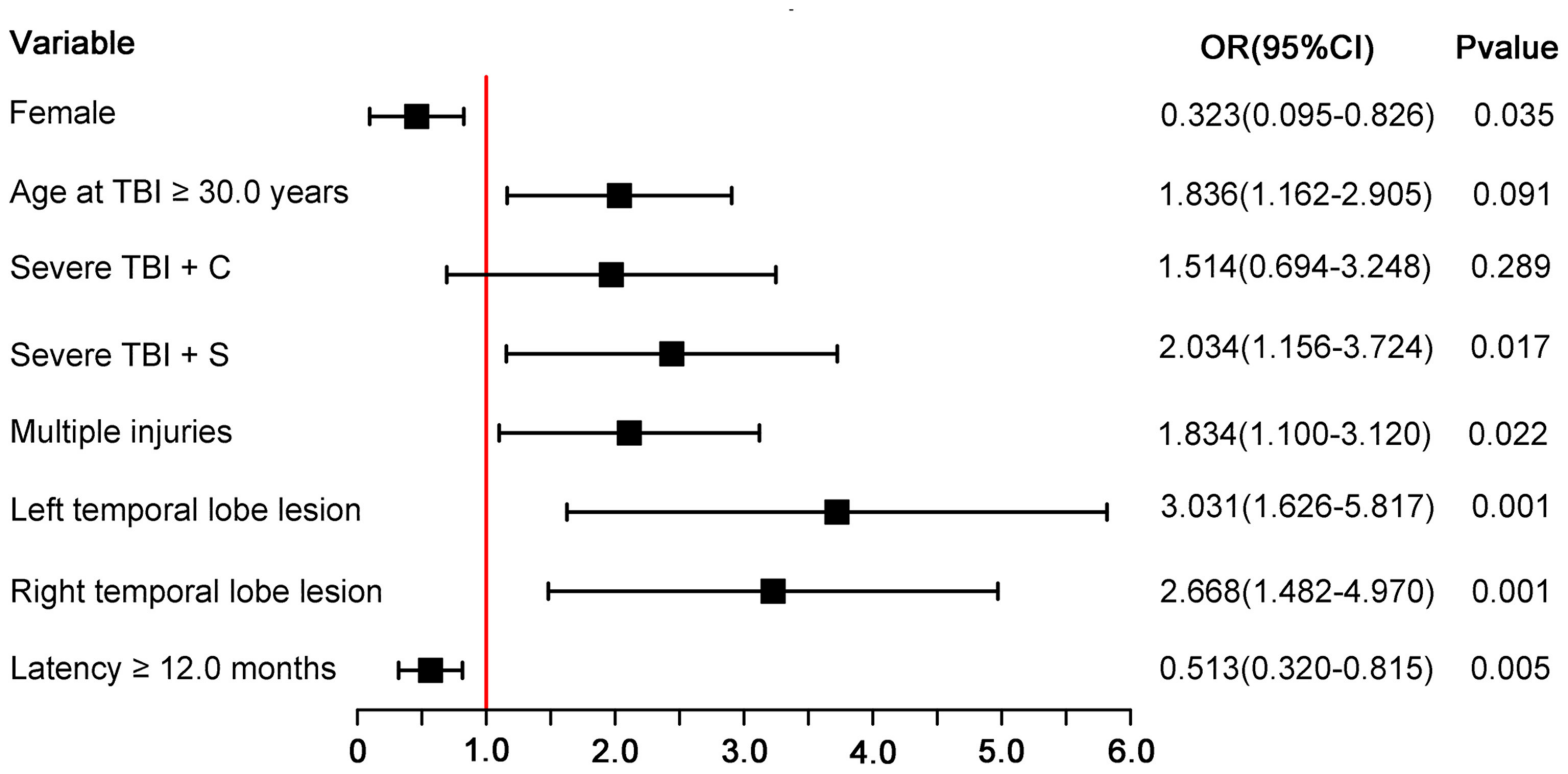
Forest plot for each predictor. The odds ratio for each predictor is presented by a square, and the confidence interval is presented by a horizontal line. Severe TBI + C, severe TBI with conservative treatment; severe TBI + C, severe TBI with surgery operation; CI, confidence intervals; OR, odds ratio.

All 6 terms in the final model were statistically significant (*P* < 0.05). Female patients were less like to have unfavorable functional outcome than male patients (OR, 0.32; 95% CI, 0.10–0.83; *P* = 0.035). Patients who had TBI at the age of 30.0 years or older were more likely to have unfavorable functional outcomes than those who had TBI younger than 30.0 years (OR, 1.84; 95% CI, 1.16–2.91; *p* = 0.009). Patients who had temporal lobe lesion were more likely to have unfavorable functional outcomes than those who had lesions outside temporal lobe (left temporal lobe: OR, 3.03, 95% CI, 1.63–5.82, *P* < 0.001; right temporal lobe: OR, 2.67, 95% CI, 1.48–4.97, *p* = 0.001). Patients who had multiple injuries were more likely to have unfavorable functional comes than those who had a single injury (OR, 1.83; 95% CI, 1.10–3.12; *P* = 0.022). Patients who suffered severe TBI with surgery operation were more likely to have unfavorable functional outcomes than those who suffered moderate TBI (OR, 2.03; 95% CI, 1.16–3.72; *p* = 0.017). Patients with a latency of 12.0 months or longer were less likely to have unfavorable functional outcomes than those with shorter PTE latency (OR, 0.51; 95% CI, 0.32–0.82; *P* = 0.005). Figure 1 is a forest plot that visualizes the results.

### Prognostic Model Development and Validation

**Figure 2:**
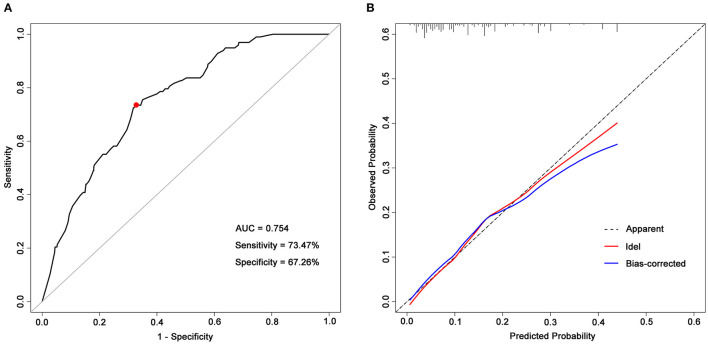
ROC curve and a calibration curve of the prognostic model. **(A)** ROC curve of the prognostic model. The prognostic model had acceptable discriminative power with an AUC of 0.754 (95% CI, 0.707–0.800). The red dot represents the optimal cutoff value, corresponding to the sensitivity and specificity of 73.47 and 67.26%, respectively; **(B)** Calibration curves of predicted probability of a 5-year unfavorable global functional outcome (*x*-axis) vs. observed probability (*y*-axis). The Hosmer–Lemeshow goodness-of-fit test was used to compare predicted probability and observed probability, p-value > 0.05 indicates good calibration. AUC, areas under receiver operating characteristic curves.

The above model demonstrated good internal validity, with a C-statistic of 0.754 (95% CI, 0.707–0.800) (Figure 2A), and the calibration curve was roughly arranged along the 45° diagonal lines, indicating good calibration as well (Figure 2B). The χ^2^ statistic of the Hosmer–Lemeshow goodness-of-fit test was 12.33 (*p* = 0.137). By using Youden's index, it was found the optimal cutoff value of the prognostic model is −1.814, corresponding to the estimated probability of an unfavorable global functional outcome of 14.0%. The sensitivity and specificity of this prognostic model were 73.47 and 67.26%, respectively. In addition, this study applied bootstrap testing to validate the model and found a C-statistic of 0.726 for internal validation.

### Presentation of the Prognostic Model

**Figure 3:**
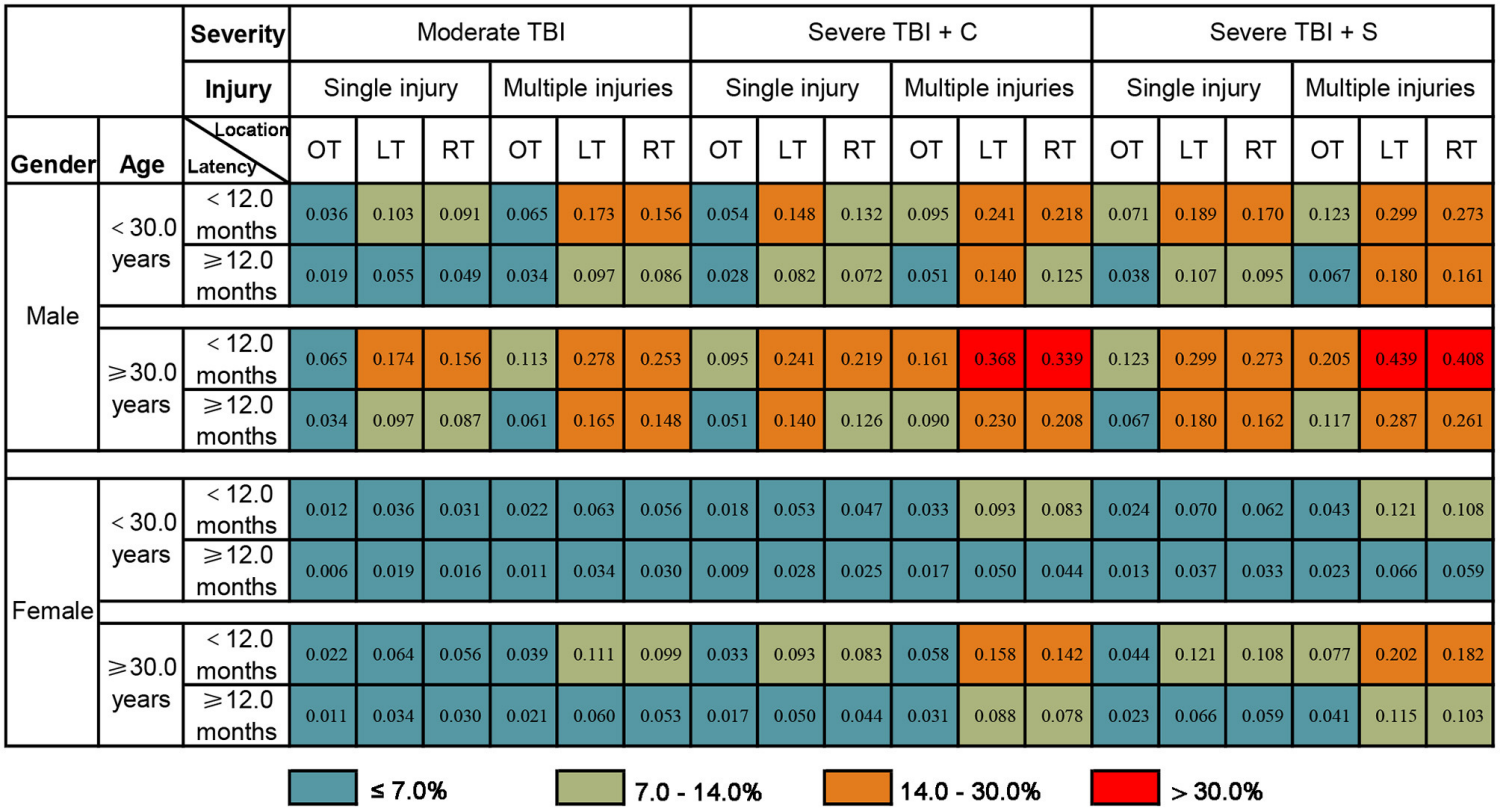
The probability of an unfavorable 5-year global functional outcome of PTE patients. The number in each box represents the probability of an unfavorable 5-year global functional outcome. Determine individual risk in two steps: Step 1, Find the corresponding cell of a given individual according to the value of each predictor; Step 2, Determine the associated risk of unfavorable 5-year global functional outcome. For example, a male patient who suffered severe TBI (single injury on the left temporal lobe) at 30.0 years old, underwent conservative treatment, with a PTE latency of 6 months. The probability of an unfavorable 5-year global functional outcome for this given patient is 24.1%. TBI, traumatic brain injury; severe TBI + C, severe TBI with conservative treatment; severe TBI + C, severe TBI with surgery operation; OT, outside temporal lobe lesion; LT, left temporal lobe lesion; RT, right temporal lobe lesion.

Taking the convenience of clinical practical application into consideration, this study presented the prognostic model as a graphical score chart in Figure 3. Here, we can directly see a patient's probability of an unfavorable 5-year global functional outcome simply by finding the corresponding cell of a given individual according to the value of each predictor. According to the cut-off value of the prognostic model, patients were divided into four categories, and Table 4 shows that observed unfavorable outcome rates of different categories matched closely with the estimated rates according to the prognostic model.

**Table 4:** The number of patients at each risk level and the observed proportion of unfavorable 5-year global functional outcomes.

**Predicted probability**	**Total (No.)**	**Unfavorable outcome (No.)**	**Observed proportion**
(0.00–0.07]	276	12	4.35%
(0.07–0.14]	175	15	8.57%
(0.14–0.30]	218	51	23.39%
(0.30–1.00]	52	20	38.46%

## Discussion

This is one of the first studies to focus on the long-term global functional outcomes over 5 years and to investigate the predictors of global functional outcomes in a sample of patients with PTE after moderate-to-severe TBI, and this study now presents the following findings. First, the global function of PTE patients assessed based on GOSE score showed dynamic changes overtime: with a favorable global function at the onset of first late PTS, 13.59% of patients experienced deterioration in their conditions and had an unfavorable global functional outcome 5 years later. Second, it was found that gender, age at TBI, lesion location, multiple injuries, the severity of TBI, and latency of PTE were predictors for 5-year global functional outcomes. Finally, a prognostic model for global function prediction by using the above variables was developed and it was presented as a graphical score chart. This model achieved significant potential to be used in clinics based on our above analysis and may help to screen patients at high risk of unfavorable global functional outcomes and to develop more effective strategies for rehabilitation.

As previously mentioned, GOSE is the recommended measurement for measuring the global function following TBI, and it outlines the overall impact of TBI on function, independence, and participation (29). To date, several studies have used GOSE for functional outcome assessment in TBI patients (5, 7, 20, 30, 31) and have suggested that there is a dynamic process of change in global functional outcomes over time, which we also found in this study.

Although the method by which gender affects the global functional outcomes remains poorly understood (32), this study found that female PTE patients were less likely to experience deterioration in a global function, and it was consistent with several previous long-term studies (20, 31) yet contrary to others (7, 33). Due to a different profession and hobbies characteristics, the TBI mechanism is different between males and females (34). Previous animal studies have shown female rodents have better outcomes after TBI than males because of the neuroprotective effect of sexual hormones (estrogen and progesterone) (35, 36); while several clinical studies also have found differences in TBI outcomes between genders, but suggesting that sexual hormones do not provide a neuroprotective effect on clinical outcomes (37, 38). Therefore, the difference in global functional outcomes between genders is still controversial, that may need to be explored in further large-sample, age-stratified, prospective studies. In addition, we realized that the influence of gender on epileptic seizure may also play a role in global functional outcomes since a larger proportion of patients were males in this present study than in previous studies (88.5% vs. 72.0–78.4%) (7, 20, 31). One other demographic characteristic, age, however, has been shown in the literature to have a clear effect on long-term functional outcomes (older patients were more likely to experience deterioration) (8, 20, 31, 39), and the findings of this present study are consistent with these earlier works. We hypothesize that the better functional outcomes of younger patients might be related not only to their physical status but also to social factors such as better medical care and better return-to-work characteristics (39).

Some authors have reported that the severity of TBI was associated with functional outcomes: the more severe the TBI, the worse the functional outcomes (39). Considering that we lacked the Glasgow Coma Scale (GCS) scores of some patients in this retrospective study, this study instead assessed the severity of TBI according to neurological and imaging evaluation (10) and found that patients who suffered severe TBI were more likely to have deterioration of global function. In addition, this study found that among patients with severe TBI, the risk of unfavorable 5-year global functional outcomes was higher in those who received surgery (OR, 2.03) than in those who received conservative treatment (OR, 1.51) compared to patients with moderate TBI. The difference may be related to the TBI condition of those patients who underwent surgery (for example, those who had marked cerebral edema, increased intracranial pressure, etc.) or to the surgical procedure itself, which may have resulted in secondary brain damage.

Multiple injuries and injuries located in the temporal lobe (especially the left temporal lobe) were also associated with unfavorable 5-year global functional outcomes. The specific role of the temporal lobe in the overall brain network may explain our findings. For most people (who are right-handed), left temporal injury is more likely to interfere with dominant-hand-motor pathways, compromise language regions, and affect language function. As language function is extremely important in independence, employment, social and leisure activities, family and friendship, and returning to normal life after TBI, we may expect patients with left temporal lobe injuries to have low long-term functional outcomes. Moreover, previous literature has also reported that epilepsy with temporal lobe damage was more likely to develop into DRE (40, 41).

We were surprised to find that, in addition to the latency of PTE, other characteristics of PTE (such as seizure type, presence of SE, drug responsiveness to ASMs, and EEG findings) were not associated with 5-year global functional outcomes. This suggests that more attention should be paid to the etiology of epilepsy, TBI, rather than to the seizure itself when assessing the functional outcome of patients with PTE. The latency of PTE ranges from 7 days to decades, with 60–80% of patients having a latency of <1 year (11, 14, 27). In this study, patients with a latency of shorter than 12 months were more likely to have deterioration of global function 5 years later, while those with longer latencies tended to have a stable global function. We realize that this result may be related to the design of the present study as it only included patients who had GOSE scores of 5–8 at their first late PTS onset. These patients who had long latencies may have already experienced a dynamic process of change in global function and already reached a stable state of global function at the onset of their first late PTS. Future prospective studies with long-term follow-up of new TBI cases and evaluation of the seizures and global function of TBI patients at different time points may help us better clarify the relationship between latency of PTE and functional prognosis.

Though it is not free from limitations (discussed below), this study has the following strengths. First, this study recorded the 5-year GOSE after the onset of the first late PTS, and the follow-up period was long enough to detect changes since the functional prognosis tends to become stable 5–10 years after TBI (6, 7). Second, this is the first study to identify the factors that affect the long-term functional outcomes among patients with PTE after moderate-to-severe TBI, establish a prognostic model, and construct a graphical score chart for clinical use, and the prognostic model can provide a more individualized prediction of the long-term functional outcomes for a patient with PTE. Specifically, based on gender, age, easily ascertainable TBI details, and latency of PTE, this prognostic model exhibited acceptable predictive capability (with a C-statistic of >0.75), and the prognostic model can be easily integrated into daily clinical practice simply by checking the graphical score chart. Once externally validated, our research may provide a basis for more effective courses of treatment.

## Limitations

There are several limitations to this study that constrain the generalizability of our findings. First, only patients with PTE were included; patients without PTE after TBI were not included as controls. The unfavorable outcomes might be mediated by the presence of PTS, rather than directly and independently correlated with factors analyzed in this study, which means that there might be selection bias that this study did not take into account. Furthermore, as this was a retrospective study, the model was developed based on factors that were recorded in the medical records or supplemented by recollections of patients or their caregivers. However, there could be other risk factors that affect global functional outcomes that need to be considered but were missed due to a lack of reliable data (such as pre-injury employment, education, etc.). This made it difficult to correct for potential confounding of variables. Finally, information bias may also exist. Further external validation is therefore needed to evaluate the prognostic model, and further large-scale prospective studies are needed to clarify fully the factors that affect the long-term functional outcomes of PTE.

## Conclusion

The global function of PTE patients assessed based on the GOSE score showed dynamic changes over time. Effective screening of high-risk patients and clearer treatment strategies are therefore essential to help ameliorate unfavorable outcomes. In this study, it was found that suffering TBI at the age of 30.0 years or older, having severe TBI (especially severe TBI with surgery), having multiple injuries, and having temporal lobe lesions were risk factors for an unfavorable 5-year global functional outcome, while being female and long (12.0 months or longer) PTE latency were protective factors. This study developed a prognostic model using these identified predictors to predict 5-year outcomes among patients with PTE after moderate-to-severe TBI, and the model achieved significant potential for clinical use. However, additional prospective studies are still needed to validate and further explore predictors of the functional outcomes of PTE patients.

## Data Availability Statement

The data that supports the findings of this study are available from the corresponding author, upon reasonable request.

## Ethics Statement

The studies involving human participants were reviewed and approved by the Ethics Committee of the Beijing Tiantan Hospital affiliated with the Capital Medical University of the People's Republic of China. Written informed consent for participation was not required for this study in accordance with the national legislation and the institutional requirements.

## Author Contributions

TY, XL, RL, and QW were major contributors to the acquisition of data. TY, LS, JW, and QW analyzed the data. TY drafted and revised the manuscript and all authors commented on previous versions of the manuscript. All authors read and approved the final manuscript. All authors contributed to the study's conception and design.

## Funding

The work was supported by the National Key R&D Program of China grant (2017YFC1307500 to QW), the Capital Health Research and Development of Special Grants (2016-1-2011 and 2020-1-2013 to QW), the Beijing-Tianjin-Hebei Cooperative Basic Research Program (H2018206435 to QW), and the Beijing Natural Science Foundation (Z200024 to YGW and QW).

## Conflict of Interest

The authors declare that the research was conducted in the absence of any commercial or financial relationships that could be construed as a potential conflict of interest.

## Publisher's Note

All claims expressed in this article are solely those of the authors and do not necessarily represent those of their affiliated organizations, or those of the publisher, the editors and the reviewers. Any product that may be evaluated in this article, or claim that may be made by its manufacturer, is not guaranteed or endorsed by the publisher.

## Abbreviations

PTE: post-traumatic epilepsy
TBI: traumatic brain injury
PTS: post-traumatic seizure
GOSE: Glasgow Outcome Scale-Extended
SE: status epilepticus
EEG: electroencephalogram
ASMs: antiseizure medications
ILAE: International League Against Epilepsy
DRE: drug-resistant epilepsy
SD: standard deviation
IQR: inter-quartile range
ROC: reiver operating characteristic curves
AIC: Akaike information criterion
AUC: areas under receiver operating characteristic curves
OR: odds ratio
CI: confidence intervals
GCS: Glasgow Coma Scale.
