# Supplementary material for: Predicting Global Functional Outcomes Among Post-traumatic Epilepsy Patients After Moderate-to-Severe Traumatic Brain Injury: Development of a Prognostic Model
Source: Front Neurol. 2022 May 30;13:874491. doi: 10.3389/fneur.2022.874491 (PMC9197334; doi:10.3389/fneur.2022.874491)
Supplement: Supplementary file 1 [file Table_1.DOCX]

**Table S1.** Multicollinearity in the final multivariable logistic regression analysis

| **Predictor variables** | **VIF** |
| --- | --- |
| Gender | 1.003 |
| Age at TBI | 1.028 |
| Lesion location | 1.090 |
| Multiple injuries | 1.119 |
| Severity of TBI | 1.061 |
| Latency of PTE | 1.039 |

*Note*: VIF < 5 demonstrates low multicollinearity

*Abbreviations*: VIF, variance inflation factor; TBI, traumatic brain injury; PTE, post-traumatic epilepsy.
