# Supplementary material for: Predicting Global Functional Outcomes Among Post-traumatic Epilepsy Patients After Moderate-to-Severe Traumatic Brain Injury: Development of a Prognostic Model
Source: Front Neurol. 2022 May 30;13:874491. doi: 10.3389/fneur.2022.874491 (PMC9197334; doi:10.3389/fneur.2022.874491)
Supplement: Supplementary file 2 [file Table_2.DOCX]

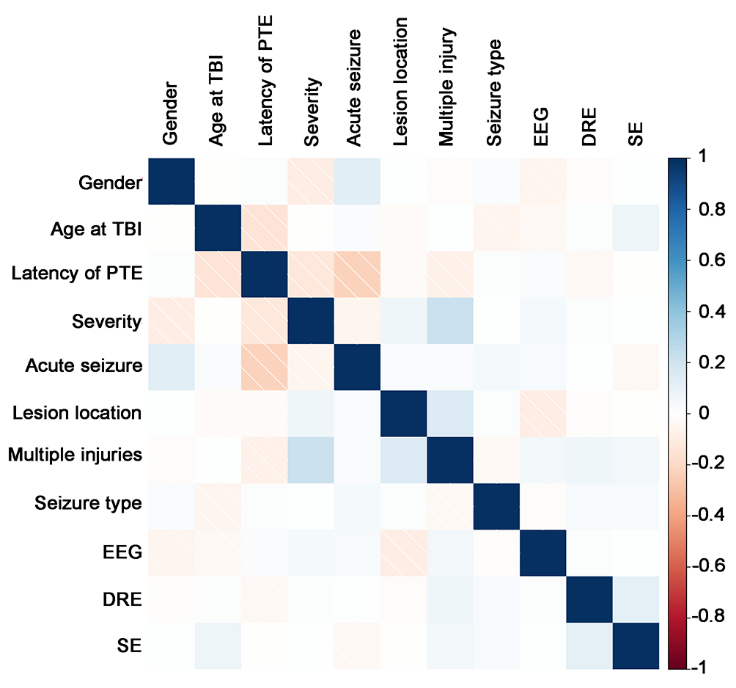


**Figure S1.** **Correlation heatmap of all variables.** Correlation analysis among 11 variables. Those variables have no correlation (absolute value of correlation coefficient < 0.3) with each other. *Abbreviations*: TBI, traumatic brain injury; PTE, post-traumatic epilepsy; EEG, electroencephalogram; DRE, drug-resistant epilepsy; SE, status epilepticus.
